# A Different SLC2A1 Gene Mutation in Glut 1 Deficiency Syndrome: c.734A>C

**DOI:** 10.4274/balkanmedj.2016.1376

**Published:** 2017-12-01

**Authors:** Rüya Çolak, Senem Alkan Özdemir, Ezgi Yangın Ergon, Mehtap Kağnıcı, Şebnem Çalkavur

**Affiliations:** 1 Clinic of Neonatology, Dr. Behçet Uz Children’s Hospital, İzmir, Turkey; 2 Clinic of Pediatric Metabolism, Dr. Behçet Uz Children’s Hospital, İzmir, Turkey

**Keywords:** Glut 1 deficiency syndrome, neonatal seizures, mutation

## Abstract

**Background:**

Glucose transporter type 1 deficiency syndrome is the result of impaired glucose transport into the brain. Patients with glucose transporter type 1 syndrome may present with infantile seizures, developmental delay, acquired microcephaly, spasticity and ataxia.

**Case Report:**

Here, we report a rare case of glucose transporter type 1 deficiency syndrome caused by a different pathogenic variant in a 10-day-old neonate who presented with intractable seizures and respiratory arrest.

**Conclusion:**

This new pathogenic variant can be seen in glucose transporter type 1 deficiency syndrome.

Neonatal seizures are rarely idiopathic and may be signs of serious central nervous system diseases. Cerebral hypoxic ischaemia, haemorrhage, hypoglycaemia, central nervous system infections, infarction, congenital metabolic diseases, central nervous system malformation and unknown factors may be considered among the most common causes (1). In the glucose transporter type 1 deficiency syndrome (Glut 1 deficiency syndrome), there is a defect in the transport of glucose from the blood-brain barrier, which is the basic fuel of for the brain (2). The first sign may be a convulsion during the neonatal period (3). While the blood glucose levels are normal, cerebrospinal fluid (CSF) glucose levels are low. Refractory epilepsy may be accompanied by growth retardation, acquired microcephaly, spasticity, and ataxia (4). In this article, a case that is clinically suspected but not supported by laboratory findings, diagnosed as Glut 1 deficiency syndrome as a result of a genetic examination, is presented.

## CASE PRESENTATION

**FIG. 1.**
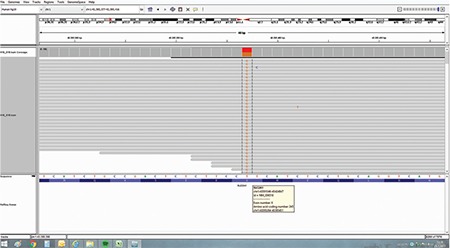
The case's *SLC2A1* gene mutation analysis image.

**FIG. 2.**
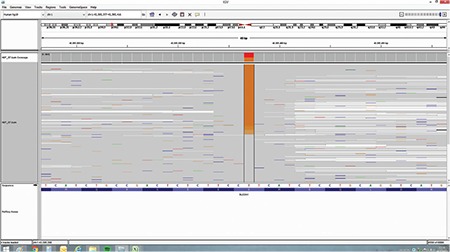
The pathogenic variant figure of patient's father.

The infant boy was the second child of non-consanguineous parents and has a healthy sister; the patient had a birth weight of 2650 grams and was taken into the neonatal intensive care unit because of resistant seizure and respiratory arrest developing on the 6th day of the postnatal period. Due to the absence of respiratory effort, the patient was ventilated with the Babylog 8000 plus ventilator (Dräger, Lubeck, Germany). Except for hypotonicity, no dysmorphic feature was determined in the physical examination. The patient had the following findings: blood pressure: 72/31 (47) mmHg, pulse: 150/min, respiratory rate: 45/min, body temperature: 36.5, oxygen saturation: 95%, and no pathological symptoms. Upon observation of hiccups and mandibular sign seizures, the patient was treated with a 20 mg/kg loading dose of phenobarbital followed by a maintenance dose of 5 mg/kg/d. The blood pressure of the case was low; therefore, dopamine and dobutamine infusion was increased gradually beginning from 5 mcg/kg/min to 15 mcg/kg/min. The case whose seizures were proceeding ha a treatment plan of a loading dose of 20 mg/kg of iv phenytoin and 20 mg/kg dose of iv levetiracetam, with a maintenance dose via iv. The case had seizures which could not be controlled, so was medicated with 100 mg of vitamin B6 via iv. As possible sepsis and meningitis would not be excluded and the general condition of the patient was not suitable for lumbar puncture, empiric treatment of vancomycin and cefotaxime was started. In terms of intracranial pathologies, cranial tomography (CT) was scanned and the CT was normal. Upon persisting seizures, midazolam infusion iv was increased gradually from 0.1 mg/kg/h to 0.5 mg/kg/h; as a result, the seizures stopped on the 12th hour of hospitalisation of the case. After stabilising the case, in terms of metabolic diseases that may cause resistant seizures, CSF biochemistry, simultaneous blood glucose, CSF amino acids, simultaneous blood amino acids, CSF culture and microscopy, tandem mass spectrometry, blood and CSF pyruvate and lactate levels, plasma sulphocysteine level, urinary sulphide test, very long chain fatty acid analysis tests were made. In the eyeground, there were no symptoms concordant with a metabolic disease. Midazolam infusion was gradually decreased and stopped. Although it was considered non-ketotichyperglycinemic in the foreground of the current Evaluation of Potency of the case, CSF glycine/glycine blood levels were normal (<0.08). In terms of glucose transporter (GLUT) defects, CSF glucose and blood glucose levels were normal (>50%), and the other metabolic investigations also gave normal results. In the case’s electroencephalography, a generalised epileptic deterioration was detected. In the cranial magnetic resonance, bilateral cerebral and cerebellar atrophy; in the ventricular system, dilatation was detected and was interpreted to show that neurometabolic neurodegenerative disease might have been a secondary development. On the 13th and 42nd days of follow-up, as the patient had 2 episodes of sepsis seizures, lumbar punctures were performed in CSF biochemistry twice more. Having a CSF glucose/blood glucose ratio of 0.69 and 1 respectively, the case could not be separated from the mechanical ventilator due to a lack of respiratory effort. Followed for 90 days with a preliminary diagnosis of severe asphyxia and metabolic diseases in the intensive care unit of newborns, the case’s peripheral blood samples were sent to the genetic laboratory in terms of GLUT defects and sequence analysis of the *SLC2A1* gene by next generation sequencing system (Miseq-Illumina - San Diego) was performed. A heterozygous pathogenic variant was found in exon 6 of the *SLC2A1* gene (NM_006516.2): c.734A>C (p.K245T) (p.Lys245Thr) (Figure 1). The pathogenic variant was detected by Intergen Genetic Centre. This pathogenic variant was not found in The Human Gene Mutation Database, ClinVar or other databases. In silico analysis results are given below (Table 1). Parental analysis showed that the patient’s father also has this pathogenic variant (Figure 2). He is asymptomatic.

After diagnosing glucose transporter type 1 deficiency syndrome (GLUTDS 1), a ketogenic diet was started. It was observed that the case, at 142 days postnatal and dependent on mechanical ventilation in the ongoing monitoring, did not have any seizures after the initiation of a ketogenic diet. Antiepileptic medications were reduced. Informed consent was taken from the patient's family.

## DISCUSSION

Glucose is one of the most essential elements for the use of brain energy. During resting, the adult brain uses more than 25% of total body glucose, while the brain of infants and children uses up to 80% (5). The blood brain barrier transport of glucose occurs by the diffusion facilitated by GLUT 1 transporter proteins. GLUTDS 1 results in no-brain glucose transportation. Classic GLUTDS 1 patients, despite multiple antiepileptic treatment during the infantile term, may present in a condition that contains complex movement disorders such as resistant seizures, growth retardation, progressive microcephaly, hypotonia, spasticity and ataxia and dystonia (5). The diagnosis is stated by 4-6 hour fasting glucose CSF/blood glucose ratio <0.4 and by the demonstration of a pathogenic variant in the *SLC2A1* gene (6). In our case, parental analysis showed that the father also has this pathogenic variant; however, he is asymptomatic. As this is an autosomal dominant disorder, it may be related to reduced penetrance or variable expressivity. Striano et al. reported a family with reduced penetrance (7). In 8 affected members of an Italian family with idiopathic generalised epilepsy, 12 manifest mainly as childhood-onset absence seizures. Striano et al. (2012) also identified a heterozygous pathogenic variant in the *SLC2A1* gene (R232C). The pathogenic variant was also found in 4 healthy adult family members, yielding a reduced penetrance of 67%. In vitro functional studies showed that the mutant protein was expressed at the cell surface but had mildly decreased glucose uptake (70%) compared to wild type. Weber et al. and Arsov et al. also reported incomplete penetrance (3,8). Seizures worsened with phenobarbital partially inhibiting GLUT 1, diazepam, methylxanthine and caffeine (9). Although having seizures that are resistant to multiple treatments, this supports the presence of hypotonia in the phenotype. In terms of laboratory findings, such as CSF glucose/blood glucose ratio being over >0.4, it has been dissociated from the GLUT 1 deficiency syndrome. Therefore, the GLUT 1 deficiency diagnosis was made after reporting the absence of an improvement of the clinical condition and would be consistent with a genetic disease. Because our case is a neonate and is constantly receiving total parenteral nutrition infusion, and CSF tests were not taken during fasting, we thought that CSF glucose/blood glucose values are normally detected. After diagnosing GLUT 1 deficiency, a ketogenic diet was started, and phenobarbital treatment was discontinued. Monitoring absence of seizures was evaluated as a response to the treatment. GLUTDS 1 has always come up with the expected disease course and laboratory findings. As for the literature, CSF glucose is >47 mg/dL and it is stated that the GLUTDS 1 diagnosis should not be ruled out in the cases where the mild phenotype is seen (10). Therefore, we would like to emphasise the importance of studying pathogenic variants which were not identified in the earlier GLUTDS 1 in the neonates who have resistant seizures and are considered to have GLUTDS 1 in terms of the course of the disease and the importance of giving the genetic counselling to the family.

## Figures and Tables

**Table 1:**
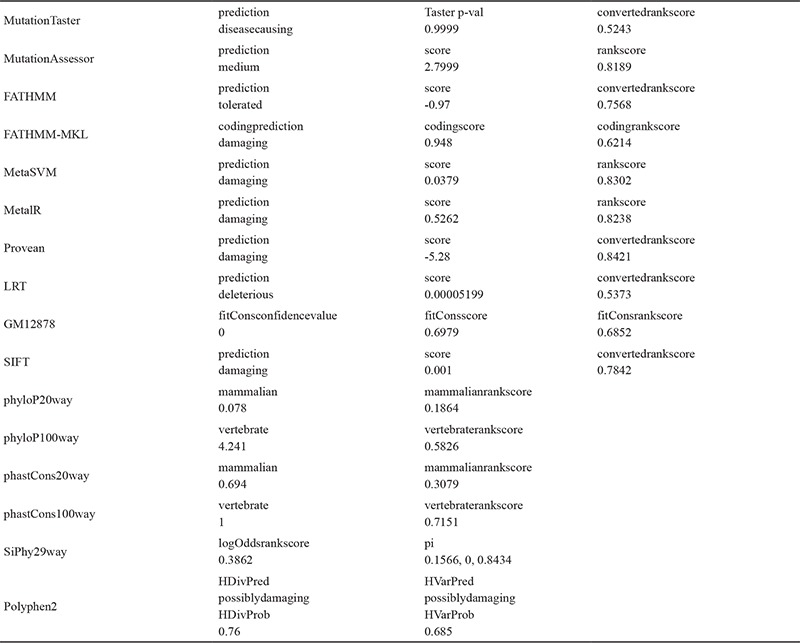
In silico analysis data of NM_006516.2: c.734A>C (p.K245T) (p.Lys245Thr) mutation
